# Mapping Information Landscapes: New Methods for Exploring the Development and Teaching of Information Literacy

**DOI:** 10.5195/jmla.2021.1136

**Published:** 2021-01-01

**Authors:** Gerald Natal

**Affiliations:** 1 gerald.natal@utoledo.edu, Raymon H. Mulford Health Science Library, University of Toledo, Toledo, OH

Andrew Whitworth is the director of teaching and learning strategy at the Manchester Institute of Education, whose research interests in information literacy include e-learning and organizational effects on learning. In an earlier work, he voiced concern that “cultural comfort and passive consumption of technologies can lead them to do a lot of our thinking for us” [1]. He provides the example of automobile drivers' reliance on global positioning system (GPS) for navigation and contrasts this with the critical judgment required to use a traditional road map.

In *Mapping Information Landscapes: New Methods for Exploring the Development and Teaching of Information Literacy*, Whitworth delves more deeply into the value of maps, which he feels are underutilized in education. He discusses information literacy in relationship to practice theory and discusses mapping as a practice with objectives similar to those of information literacy: namely, finding, selecting, organizing, and communicating information.

The book provides major examples of the sociopolitical influence of maps, from cartography to the unconventional, and relevant theories of information, communication, and practice are used to, in his words, “map the field of mapping” (p. 170). Attention is drawn to the similarities between the physical and virtual worlds—between geographical and informational landscapes—and the ways in which both are navigated. The concepts of “space,” “place,” and “time” are emphasized and featured throughout as essential but, heretofore, unexplored elements of information literacy. Mars is used as an intriguing example of how a “space” becomes a “place” through mapping. A considerable amount of text is dedicated to illustrating how maps are defined by and used to exercise power and authority, but also to empower users.

Whitworth devotes three chapters to autoethnographic, self-reflexive, and qualitative research to underscore main concepts. One major concern is how information literacy translates outside of the limits of higher education. The investigated studies occur in both formal and informal educational settings, and study results are used to make connections between *place, affect, practice*, and effective judgment and to demonstrate how assessment is used to establish power relationships with students.

The processes of routine, embedded memory, observation, and general assumption are connected to learning and information literacy. Students' information-seeking behavior is explored, while the benefits of content mapping to information literacy are brought to light. Other research focuses on discursive mapping, the process of mapping, and its impact on learning. To support this detailed examination, Whitworth refers to names and concepts that are familiar to readers in the areas of information literacy and education (e.g., Elmborg, Freire, Foucault, Badke, and Kulthau: “Six Frames for Information Literacy Education” [2]) as well as influences from communications, psychology, and other relevant disciplines.

*Mapping Information Landscapes* is for any reader who is interested in information literacy, mapping as an educational approach, critical theory, or maps in general. Librarians will find themselves reminded that they are authority figures in sociotechnical systems, as “product[s] of professional context” (p. 7). The book should encourage librarians' active involvement and a repositioning of themselves as facilitators, as opposed to teachers. Whitworth makes the case that information literacy education needs to transcend the boundaries of the library—from teaching to enabling to informed learning. It is an enjoyable as well as an educational read that sets the groundwork for further inquiry.
